# Self-esteem in sexual minority young adults: a qualitative interview study exploring protective factors and helpful coping responses

**DOI:** 10.1080/09540261.2022.2051446

**Published:** 2022-04-23

**Authors:** Livia Bridge, Patrick Smith, Katharine A. Rimes

**Affiliations:** Department of Psychology, Institute of Psychiatry, Psychology & Neuroscience, King’s College London, London, UK

**Keywords:** Resilience, confidence, youth, LGBT, qualitative, self-worth

## Abstract

Research on mental health inequalities between sexual minority and heterosexual young adults has historically focussed on the additional stress processes that might explain this disparity. However, more recently there has been a shift towards research focussed on resilience factors that might promote mental health in sexual minority young adults. Self-esteem is one such proposed resilience factor. This study aimed to explore the factors that promote or protect self-esteem itself in sexual minority young adults. A semi-structured interview study was conducted with 20 sexual minority young adults (aged 16–24) to explore their perspectives on the factors, responses and strategies that have helped to protect or promote their self-esteem. Six themes were identified from thematic analysis: helpful responses to minority stress; sexuality acceptance; positive LGBTQ + social connections and representations; positive social relationships and evaluation; successes and positive qualities and general coping strategies for low self-esteem. Findings are discussed in terms of their theoretical implications.

## Introduction

Mental health inequalities based on sexual orientation are well-established in young adulthood (Birkett et al., 2015; Semlyen et al., 2016). Minority stress models have tended to focus on the additional stress processes that might explain this disparity (Meyer, 2003) but there has been a shift towards acknowledging the importance of research focussed on resilience factors for mental health in this population (Kwon, 2013; Meyer, 2015). LGBTQ + resilience can be defined as the ability to persevere or flourish in the face of harassment, rejection, discrimination or oppression based on sexual orientation or gender identity (Meyer, 2015). Positive self-esteem is a potential resilience factor in this context. However, little research has focussed on the mechanisms that help to promote or protect self-esteem itself in this population.

Coping and resilience in relation to self-esteem are especially important in the context of young adulthood, a key period of social identity development, where having a stigmatized sexual orientation brings unique challenges. Sexual minority young adults need to cultivate ways of coping with minority stress related to their sexual orientation in addition to building resilience for general life stressors. To date, few studies have directly examined the impact of coping responses on self-esteem in sexual minority young adults.

One quantitative study found that self-rated use of LGBTQ + specific responses to minority stress, but not general cognitive strategies, were significantly associated with more positive self-esteem in sexual minority young adults (Toomey et al., 2018). A recent qualitative study also found that minimizing the importance of the discriminatory individual and distinguishing opinions of others from facts helped protect self-esteem from minority stress for some sexual minority young adults (Bry et al., 2018). However, no previous studies have specifically explored the potential factors or strategies that help to maintain or promote positive self-esteem from the perspective of sexual minority young adults. This study aimed to address this gap in the literature by exploring the factors or strategies that promote or protect self-esteem in sexual minority young adults using a qualitative semi-structured interview design.

## Method

### Participants

Participants were young adults aged 16–24 that self-identified as having a minority sexual orientation. Full inclusion and exclusion criteria are described in detail in the accompanying paper (Bridge et al., in press).

### Measures

Measures of self-esteem, anxiety, depression and self-criticism were collected before each interview to help characterize the psychological characteristics of the sample in relation to population or clinical norms. These have been described in the accompanying paper and were: Rosenberg Self-Esteem Scale (Rosenberg, 1965), Patient Health Questionnaire (Kroenke et al., 2001), Generalized Anxiety Disorder Questionnaire (Spitzer et al., 2006) and Forms of Self-Criticizing/Attacking & Self-Reassuring Scale (Gilbert et al., 2004).

### Procedure

Ethical approval was granted by the relevant research ethics subcommittee (Ref: HR-17/18-5266). Purposive sampling was used to recruit sexual minority young adults with a range of different self-esteem levels, sexual identities, ethnicities and gender identities. This study was part of a larger qualitative project (Bridge et al., in press), with the same participants, which also investigated factors having a negative impact on self-esteem. The study was primarily advertised online on social media and through a UK research participation recruitment website. Participants who registered an interest in the study were sent a participant information sheet via email. After screening eligible participants were then invited to attend an interview (details are provided in the accompanying paper).

Each interview followed a semi-structured interview schedule outlining topics and broad questions to be explored with suggested prompts for further detail. Questions in the interview guide were developed based on review of the literature and gaps in the current evidence base, with feedback from sexual minority young people. Questions covered the following broad areas: (1) experiences of sexual orientation-related stigma, prejudice or discrimination, (2) protective and positive factors/experiences for self-esteem and (3) coping strategies or responses for low self-esteem. Questions were open-ended, which allowed participants to discuss and focus on experiences that they believed to be important and relevant.

### Data analysis

Interviews were coded and analysed using the steps and principles of thematic analysis (Braun & Clarke, 2006). Analysis followed Braun and Clarke’s (2006) six-step guidelines, from coding to theme refinement. The first five interviews were transcribed by the first author to aid familiarization with the data. Remaining audio-recordings of the interviews were transcribed verbatim by a professional external transcription service. Next, line-by-line coding was completed for five of the twenty interviews. Initial coding took place before final recruitment to ensure theme saturation had been reached. Initial codes were collated into potential themes and subthemes to create a working ‘thematic map’. Line-by-line coding for the remaining 15 interviews was then conducted and any new codes were added to the ‘thematic map’. Themes were refined several times throughout this process where new codes were identified, and higher order themes were developed. Theoretical and conceptual credibility checks were carried out by the researcher’s primary and secondary supervisors several times during the refinement period. When final themes were agreed on, final name-headings and summaries for each theme and sub-theme were generated and again checked by the primary and secondary supervisor for validity.

## Results

### Participant characteristics

Participants were the same sample described in the accompanying paper (Bridge et al., in press). Participant characteristics and scores on questionnaire measures are summarized again in Table 1.

**Table 1.** Participant characteristics and scores on psychological measures.

Participant characteristics		
Age (SD)	20.1 (2.3)	
	N (%)	
Gender identity		
Man	7 (35)	
Woman	12 (60)	
Non-binary	1 (5)	
Sexual orientation		
Lesbian	6 (30)	
Gay	3 (15)	
Bisexual	7 (35)	
Pansexual	2 (10)	
Queer	1 (5)	
Asexual/bi-romantic	1 (5)	
Ethnicity		
White British	6 (30)	
Black	3 (15)	
Asian	4 (20)	
Other White	3 (15)	
Mixed background	4 (20)	
Measures	Mean (SD)	Range
RSES	26.1 (5.8)	18–33
FSCS		
IS	21.1 (9.2)	1–34
HS	16.2 (7.2)	0–15
RS	5.3 (4.7)	4–29
GAD-7	6.9 (4.4)	0–14
PHQ-9	7.4 (5.2)	0–17

RSES: Rosenberg Self-esteem Scale; FSCS: Forms of Self-Criticizing/Attacking and Reassuring Scale (IS: inadequate self; HS: hated self; RS: reassuring self); GAD-7: Generalized Anxiety Disorder-7 Questionnaire; PHQ-9: Patient Health Quesitonnaire-9.

### Factors, strategies or responses that protect or promote self-esteem

All participants reported that they had at some point in their life experienced external stigma, prejudice, discrimination or stress, related to their sexual orientation, as well as general life stressors. Both are described in more detail in the accompanying paper (Bridge et al., in press).

All participants discussed a range of factors, strategies or coping responses that they felt had helped to either promote positive self-esteem or had protected their self-esteem from the potential negative impact of the stressors discussed above.

Six key themes were identified from thematic analysis: (1) helpful responses to minority stress, (2) sexuality acceptance, (3) positive LGBQ + social connections and representation, (4) positive social relationships and evaluations, (5) successes and positive qualities and (6) general coping strategies for low self-esteem. The first three themes describe sexuality specific factors/coping strategies, whilst the latter three themes pertain to more general factors/coping responses.

Note: Each quote is labelled with participant details, namely gender (M/F/NB) for man, woman and non-binary respectively, sexual identity (G, L, B, O) for gay, lesbian, bisexual and other, respectively and ID number.

### LGBTQ + specific factors: helpful responses to minority stress

This theme captured responses to sexual orientation-related stigma or discrimination that the young adults felt had helped them to maintain positive self-esteem. More active behaviours included activism and education, whereas others described internal cognitive strategies such as shifting blame and devaluation of perpetrators or empathy for themselves and others.

Participants believed that involvement in activism helped improve their self-esteem as they felt it gave them purpose, or that their minority sexual orientation could have a positive influence. For some, being more educated themselves about LGBTQ + experiences helped them feel more secure or justified that their own views on sexuality were correct and allowed them to devalue negative attitudes of others, e.g.

Becoming more educated myself and also reading other people’s experiences online or talking to other people about it and just discussing it to get a more overall view has been good because the more secure I feel in that, that my view of the thing is the correct one then it won’t affect me at all. (MB5)

For some, they felt that they were not responsible for the attitudes of others, which had protected their self-esteem after negative reactions towards their sexuality:

So, it’s like, if I can blame her for it, then it takes, like, the negative feelings off myself. It’s helpful in the sense that it makes me feel better about myself, because I don’t feel like I’m at blame for whatever it is that I, almost feel guilty for. (FB9)

Alternatively, empathy towards themselves or others after experiences of minority stress was discussed by some young people here as helping to reduce the negative implications for their social identity and self-evaluation. Participants described a process where they were sometimes able to understand the actions of others and reframe the reason for the prejudicial attitudes as external, rather than indicating the perpetrator did not value them at a personal level. Empathy towards others also appeared to be an important buffer for self-esteem in sexual minority young adults when perpetrators were close others, because devaluing the perpetrator might not be possible or might negatively impact an important social relationship.

### LGBTQ + specific factors: sexuality acceptance

This theme captures how young adults’ acceptance of their own sexuality helped to improve their self-esteem. This included feeling that their sexuality was a positive quality, embracing every part of themselves or feeling secure in their sexual orientation. For some, they also felt that self-acceptance of their sexuality had helped to prevent the negative impact of stigma-related stressors on their self-esteem e.g.

[it would impact my self-esteem] if I wasn’t so secure in knowing that loving who you want to is completely fine, like that to me isn’t an evidence or experienced based thing. That is something I just intrinsically know to be the case and nothing that anyone says or does will ever convince me otherwise. (FL08)

Several participants felt that telling other people about their sexuality had helped them to accept their own sexuality, e.g. ‘starting to tell people, was definitely a point in which I started to, like, accept myself and now, like, being gay is something I absolutely love’ (FL08). Telling others about their sexuality allowed overt positive feedback or acceptance from others which one participant discussed had helped facilitate greater self-acceptance, e.g. ‘When I started to be more open about my sexuality and saw how people, sort of, accepted it, that made me feel less bad about being gay’ (MG16). Some participants also believed that being open about their sexuality had prevented some overt homophobia or discrimination that they believed might otherwise have affected their self-esteem, for example, for one participant ‘knowing that I’m gay means [friends] don’t want to say anything stupid, or anything homophobic’ (FL20).

### LGBTQ + specific factors: positive LGBTQ + connections and representation

This theme identifies the beneficial or protective impact on self-esteem of positive connections with others in relation to sexual orientation and the importance of having sexual minority role models and representation. These experiences were felt to both protect self-esteem from perceived stigma or discrimination and promote positive feelings about sexual orientation.

Most participants reported that support from other sexual minority individuals provided them with a more general sense of belonging and lessened feelings of being different or out of place, e.g.

I never, kind of, felt any less or any more for being bisexual, but I think that’s just ‘cause I had enough people around me who were the same, do you know? You know, like, enough people around me who were gay or lesbian or bisexual or very open about supporting any of the above, to kind of feel included somewhere. (FB10)

Having a supportive friend with shared experiences and understanding to talk to, was reported to have helped prevent self-critical rumination about minority stress experiences. Being part of a support network of friends that discussed the shared view that sexual orientation-related stigma or discrimination is wrong, also provided greater confidence and empowerment to feel angry rather than be self-critical. Further, for some of the sexual minority young adults here, relationships with others who had similar experiences related to their sexuality, helped normalize their own experience and promote acceptance of themselves. Young adults described feeling that because they were able to love and accept their sexual minority friends, they in turn felt more able to love themselves, for example, ‘Like, if I’m reassured by other people who are in a similar position to me, it just makes me feel like I’m not wrong’ (FB9).

Half of the participants said that feeling part of an LGBTQ + community helps to protect self-esteem from stigma processes and promoted positive feelings about their sexuality. This sense of community connection could be at a personal level or a broader institutional or societal level.

Positive representation of sexual or gender minority people, their rights and relationships, was also suggested to have positively impacted self-esteem for several young adults. They discussed how, for them, more positive representation helped them to see that having a minority sexual orientation is acceptable and reduced shame around it as ‘it’s not exclusively straight relationships you have portrayed, like in music as well there’s more space for like LGBTQ people … that can show you that it’s not anything to be ashamed of’ (MB5). Positive reactions in the media also helped some to feel more positively about their own sexual orientation. For example, for one young person, reading ‘a lot about, like, stories of, LGBT couples it makes me feel like I’m represented… I think it’s what keeps me going. Because I can see that people enjoy them as well, it makes me feel like they’re enjoying me’ (FB15).

Similarly, participants spoke about the positive impact that role models had on their self-esteem, especially real-life role models as opposed to only celebrities. Having role models who had successful life experiences provided them with optimism for their own future, whilst for others, role models who they admired or respected helped them to accept their own sexuality, e.g.

I feel like that its quite beneficial to self-esteem, because I feel like if [sexual minority Youtubers] are quite good in themselves and they’ve got a relationship and they’re happy, they’ve got a job and stuff like that, you just feel like, well, you know, we’re the same people – and they’ve been able to have a positive life and enjoy their life. (MG11)

### General factors: positive social relationships and evaluations

This theme illustrates how positive general social relationships and perceived positive feedback from others promoted feelings of self-worth. Almost all participants discussed feeling that close social relationships helped them to feel loved and valued by others. Several young adults here felt they were able to internalize the love or value they experienced from others towards themselves to strengthen positive self-evaluations because ‘having people love you can help you love yourself’ (MG6). Several participants described how relationships had helped them to develop their own self-worth. This could be through more specific positive beliefs about themselves such as being ‘loveable’ or ‘worth something’ or a more general positive sense of self, e.g.

I’ve always had, like, really good friends. I was very lucky in that and I think that definitely positively impacted my self-esteem because, yeah, it made me feel, like, good about myself, ‘cause they loved me, like, unconditionally. (FL12)

Several participants also felt that love and value from close others could be used to help reassure themselves in response to self-critical thoughts after stressful experiences in order to boost their self-esteem. For example, for one participant:

If you just have someone constantly telling you positive things and, like, reassuring you, then – at one point they, kind of, like, go in. I guess if someone loves you, then you’re like, oh, like, I am good. I am worth loving. (FL12)

In a similar but distinct process, some young adults also discussed how they felt that evidence that they were generally well-liked and respected amongst their peers, or ‘popular’, could boost their self-esteem which again reflects broader acceptance. Evidence of popularity led the young adults to reflect on their social value or status and feel more positively about their own characteristics or qualities. Evidence of social standing or value to others for some was gained through their notoriety, number of friends and/or positive social feedback. Several participants discussed how feeling generally ‘appreciated’ or ‘liked’ by others had boosted their self-esteem. Being valued by others helped some participants to reflect on their positive qualities e.g. ‘if you feel like other people value you, then you might start to think, okay – what do they see in you, you know, what’s special about me?’ (MG6).

Appreciation and praise from parents and teachers allowed them to reflect on and strengthen their own more positive self-evaluations but related mainly to competence. For example:

I suppose like my parents and my mum was quite good about boosting me up and making me feel good about myself and … she would always say that like ‘I’m so clever’ or stuff like that … it still always made me feel better. (MB5)

### General factors: successes and positive qualities

Almost all participants reported that their achievements or successes in various domains positively impacted their self-esteem. Most participants spoke about self-esteem being positively ‘correlated to, sort of, feeling of success in whatever I am trying to do’ (FL18) and that feelings of positive self-worth ‘come from successes and stuff like empirical evidence’ of ‘succeeding’ (FL8). Participants reported feeling ‘accomplished’ when ‘content with what [they have] achieved’ (FB13). Several participants also reported that their success or achievements impacted their self-esteem through ‘validation’ from respected others.

Several participants spoke about having ‘interests’, ‘talents’ or ‘abilities’ that contributed to more positive thoughts and feelings about themselves and higher self-esteem. Talents or abilities discussed by participants included academics, sport and music, as well as more specific interests. Having a particular ability or talent was reported to boost self-esteem as ‘it makes me feel like I’m good at something. It makes me feel like there’s something special about me.’ (MG6)

Participants talked about their abilities having a positive impact on self-esteem through reflecting on what it implies about them as a person, for example, one participant said, ‘if I, sort of, self-reflect on who I am and what I am and what I can do, so, like, in terms of sports and in terms of like academics or studying then I feel really good about myself’ (FO3).

Some participants also believed that feeling ‘productive’ was linked to higher levels of self-esteem. Several young adults discussed doing activities deemed as productive could help boost mood and consequently self-esteem when feeling negative e.g.

if I was, like, at home feeling like **** and then I’d maybe, like, play guitar, or like, even get out of bed and have a shower, like, maybe even go out and see people, you know, like doing productive things, then that makes me feel a lot better about myself. (FB10)

Similarly, contributing to prosocial activities such as volunteering and more generally supporting friends and family were identified by several young adults as having a positive impact on their level of self-esteem as they could see that their actions provided a sense of purpose, e.g.

I’m working in a disability centre … it’s also helping me at the same time, it makes me feel better about myself, that I’d helped others in need and that I’m actually helping other people in the community and that I’m not that, like, insignificant. (FB13)

### General factors: coping strategies for low self-esteem

This theme captures strategies or responses to negative thoughts or feelings about the self. Strategies directly addressed negative thoughts or helped to distance or distract from them. Almost half of participants discussed that their self-esteem had improved after having received some form of psychological therapy such as cognitive behavioural therapy, mindfulness and counselling. Although the therapies received were not specific for self-esteem, some participants felt that therapy had been ‘the most helpful thing for my self-esteem’ by giving them tools to challenge negative thoughts about themselves. For example, trying to ‘derail the argument by working out a loophole in it’ (NB2). However, some participants had mixed experiences of therapists, one participant felt that their therapist had either been ‘a little homophobic’ (FL08) or not specifically educated on experiences relevant to queer or ethnic minority individuals.

Some participants described using similar techniques to challenge their negative thoughts about themselves, independently of psychological therapy. Alternatively, several participants spoke about how being ‘kinder’ to themselves, learning to ‘love’ themselves, reciting ‘positive affirmations’ and trying to ‘not be so hard’ on themselves had helped to improve their self-esteem or sense of worth as ‘It just helped me being, like, kinder to myself and, therefore, like, feeling better about myself’ (FL12). Some participants also felt that being kinder could help reduce their more self-critical thoughts, e.g.

it’s, like, a lot of like, loving yourself and, it’s okay if you have these thoughts… not being so hard on yourself… I think that was one of my issues, was just being really hard on myself and made me feel more negative. (FL12)

Almost all participants spoke about using ‘distraction’ as a way of coping with negative thoughts about themselves and for some, raising their self-esteem. For most young adults, they believed that distraction helped their self-esteem by disengaging from negative thoughts about themselves. For some participants, they also discussed how having a creative outlet for their negative thoughts about themselves had helped to improve or cope with their low self-esteem. Creative outlets here were used to process negative thoughts rather than as a distraction.

## Discussion

This study suggests that general factors or coping responses, as well as sexual minority specific ones, might be important for self-esteem resilience in sexual minority young adults. This finding differs from a previous retrospective study of coping responses in sexual minority young adults, which found a positive correlation between sexual minority specific but not general coping responses and higher self-esteem (Toomey et al., 2018). Participants discussed how being kinder or more compassionate to themselves in response to or instead of self-critical thoughts helped to improve their self-esteem. This supports previous evidence of an association between self-compassion and self-esteem in LGBQ + individuals (Beard et al., 2017). Further, sexual minority young adults here highlighted the importance of their own successes and positive qualities in non-interpersonal domains for their self-esteem. Higher investment of self-worth in other domains is a potential compensatory strategy in the face of adverse interpersonal experiences (Pachankis & Hatzenbuehler, 2013).

The current study also adds to the literature on potential resilience factors within minority stress models of sexual minority mental health inequalities (Meyer, 2015). Hatzenbuehler’s (2009) psychological mediation framework proposes that the impact of minority stress on mental health is partly mediated by the negative effect on general mental health risk factors such as self-esteem. Participants discussed both cognitive and behavioural responses to their experiences of minority stress that they felt had either prevented the experience from affecting their self-esteem negatively or had helped to improve their self-esteem. Specific positive coping responses identified here could be added to the broad ‘coping strategies’ element of minority stress models which do not currently specify these responses. This could include educating self/others, activism, empathy for self and others, and devaluing/blaming perpetrators of stigma or discrimination.

Current findings also highlight sexual minority specific factors that might help promote self-esteem not as a direct response to minority stress, but that may build self-esteem resilience. Higher self-esteem in general might then, in turn, dampen the impact of stigma-related stress on mental health outcomes. Our findings suggested that self-acceptance might protect self-esteem from minority stress experiences. Participants felt that when they accepted their sexual orientation, they were able to dismiss the relevance and potential adverse impact of stigma or discrimination to their self-evaluation. Findings here provide more detail about the mechanisms underlying previous research demonstrating findings of an association between sexuality self-acceptance and self-esteem (Woodford et al., 2014; Rostosky et al., 2018).

Disclosure of their sexuality to others contributed to self-acceptance for our participants, where disclosure and consequent acceptance of their sexuality by others helped them to accept their own sexuality. This finding is in line with Elizur and Mintzer’s (2001) sexual identity development theory, which outlines that self-acceptance is one of three interlinked processes experienced by sexual minority individuals, alongside building a self-definition, and disclosing their sexuality to others. Participants described a process where they felt loved and accepted by others after receiving positive responses to sexuality disclosures. In turn, they then felt that if others loved and accepted them for who they were, they were able to internalize this to love and accept themselves.

Social support provided by LGBTQ + friends was suggested by most of the sexual minority young adults to protect their self-esteem from the negative impact of stigma-related stressors. This finding supports previous qualitative and experimental research consistent with the possibility that social support acts as a buffer or resilience factor against the negative impact of stigma-related stressors (Burton et al., 2014; Bry et al., 2018) but extends this to self-esteem in sexual minority young adults.

As well as personal friendship support, about half of the participants mentioned the importance for their self-esteem of a sense of belonging to a wider LGBTQ + community. Firstly, being part of the LGBTQ + community had helped them to feel included and less like they were different, by providing a place where they felt that they fitted in. This is consistent with social evolutionary theoretical perspectives of self-esteem, especially sociometer theory, which propose that self-esteem reflects our internal subjective sense of how valued we are by others where level of inclusion is an important indicator of this (Leary et al., 1995). Finding that a sense of LGBTQ + community belonging had a positive impact on self-esteem is also consistent with previous evidence in other stigmatized groups that in-group identification and connections within their stigmatized social group is associated with more positive self-esteem (Bat-Chava, 1994; Rowley et al., 1998). It is also consistent with previous cross-sectional research showing that connectedness to the LGBTQ + community is associated with reduced self-criticism in sexual minority young adults (Puckett et al., 2015).

The importance of cultural representation and sexual minority role models for self-esteem was also reported by these young adults. Previous cross-sectional research has found evidence that identification with role models during adolescence for stigmatized individuals, including sexual minority young people, is associated with more positive mental and physical health outcomes, including positive self-worth (Bryant & Zimmerman, 2003; Grossman & D'Augelli, 2004). The current study provides evidence for potential mechanisms (optimism and self-acceptance) through which having role models promotes self-esteem.

## Limitations

Although efforts were made to recruit a representative sample of sexual minority young adults, the majority were still white and attending higher education. However, with a qualitative interview design, it is possible that individuals’ responses might not fully reflect their own thinking processes and behaviours, especially those for which they are less aware or reluctant to report. Further, as with any qualitative analysis, interpretations of themes should be seen as preliminary and further exploratory work is needed to confirm the direction of the relationships discussed here. For example, higher levels of self-esteem might have meant that participants were more able to use certain coping strategies, perceive or retrieve positive experiences associated with self-esteem, and were more likely to receive positive feedback from others. Participants were not asked about this. It is difficult to disentangle these relationships within a qualitative interview design without leading the interviewee. Some reciprocal effects might also be less obvious to participants and therefore less likely to be mentioned spontaneously in an interview without explicit prompting and require further investigation using both qualitative and quantitative research designs.

## Conclusions

This study explored the factors, strategies or responses that help to protect or promote self-esteem in sexual minority young adults using semi-structured qualitative interviews. Findings showed that both sexual minority specific and more general coping responses to minority stress and general life stressors helped to protect self-esteem in this population. General factors or strategies included those often used in psychological therapies, such as thought challenging and self-compassion, as well as social relationships, and noting their own successes or positive qualities. Several sexual minority specific strategies or factors were identified here, including helpful cognitive/behavioural responses to minority stress experiences, sexuality acceptance and positive LGBTQ + experiences, that have implications for minority stress and resilience models.
